# Unbiased MRI Analyses Identify Micropathologic Differences Between Upper Motor Neuron-Predominant ALS Phenotypes

**DOI:** 10.3389/fnins.2019.00704

**Published:** 2019-07-12

**Authors:** Venkateswaran Rajagopalan, Erik P. Pioro

**Affiliations:** ^1^Department of Electrical and Electronics Engineering, Birla Institute of Technology and Science, Pilani, Hyderabad, India; ^2^Department of Biomedical Engineering, ND2, Lerner Research Institute, Cleveland Clinic, Cleveland, OH, United States; ^3^Department of Neurology, Neuromuscular Center, Neurological Institute, Cleveland Clinic, Cleveland, OH, United States; ^4^Department of Neurosciences, Lerner Research Institute, Cleveland Clinic, Cleveland, OH, United States

**Keywords:** MRI, UMN-predominant ALS, corticospinal tract hyperintensity, diffusion tensor imaging, micropathologic differences, fractal dimension, ALS phenotypes

## Abstract

Amyotrophic lateral sclerosis (ALS) is an incurable and progressively fatal neurodegenerative disease that manifests with distinct clinical phenotypes, which are seen in neuroimaging, and clinical studies. T2- and proton density (PD)-weighted magnetic resonance imaging (MRI) displays hyperintense signal along the corticospinal tract (CST) in some ALS patients with upper motor neuron (UMN)-predominant signs. These patients tend to be younger and have significantly faster disease progression. We hypothesize that such ALS patients with CST hyperintensity (ALS-CST+) comprise a clinical subtype distinct from other ALS subtypes, namely patients with UMN-predominant ALS *without* CST hyperintensity, classic ALS, and ALS with frontotemporal dementia (FTD). Novel approaches such as fractal dimension analysis on conventional MRI (cMRI) and advanced MR techniques such as diffusion tensor imaging (DTI) reveal significant differences between ALS-CST+ and the aforementioned ALS subtypes. Our unbiased neuroimaging studies demonstrate that the ALS-CST+ group, which can be initially identified by T2-, PD-, and FLAIR-weighted cMRI, is distinctive and distinguishable from other ALS subtypes with possible differences in disease pathogenesis.

## Introduction

Etiology and site of origin of amyotrophic lateral sclerosis (ALS) within the central nervous system (CNS) are unknown (Mitsumoto et al., 1998). ALS diagnosis is based on motor neuron degeneration in both the CNS and peripheral nervous system (PNS), which include the upper motor neuron (UMN) and lower motor neuron (LMN), respectively. However, whether ALS begins in the CNS (Eisen et al., 1992) or PNS (Chou and Norris, 1993) is debated. Even if we consider a CNS origin, precisely where degeneration begins along the UMN pathway is unknown, as it can be anywhere along its rostrocaudal extent. If pathology originates in the corticomotoneuron within the cerebral cortex it would be considered a “neuronopathy”; if it originates somewhere along the axon in motor tracts (e.g., corticospinal and corticobulbar) within the subcortical white matter or spinal cord, it would be considered an “axonopathy.” If ALS is an axonopathy, degeneration would begin distal to the neuronal cell body, and proceed retrogradely to affect it later; if it is a neuronopathy, the neuronal cell body would be affected first with subsequent loss of the entire axon because of wallerian degeneration.

In previous studies, we have evaluated brain MRI changes in patients with ALS based on their clinical phenotype and extent of UMN or cognitive impairment, including in those with UMN-predominant ALS, classic ALS [expressing relatively equal amounts of UMN and lower motor neuron (LMN) dysfunction], or ALS with frontotemporal dementia (ALS-FTD). Although an ALS diagnosis relies on the clinical presence of both UMN and LMN signs, a proportion of patients with ALS present with evidence of only UMN abnormalities and develop LMN signs later. A hyperintense signal is visible along both corticospinal tracts (CST’s) on conventional T2-, proton density (PD)-, and FLAIR- weighted MRI in some patients with predominant or exclusive UMN signs (Mitsumoto et al., 1998), while others do not (Matte and Pioro, 2010), even though both patient groups have comparable degrees of clinical UMN dysfunction. A review of the literature revealed 17–67% (median 40%) of ALS patients with CST hyperintensity (Pioro, 2006), while a preliminary analysis at the Cleveland Clinic found this change in ∼30% of ALS patients (Matte and Pioro, 2010). Although the precise cause of CST hyperintensities is unknown, an early radiologic-histopathologic study showed demyelination and wallerian degeneration in fibers of the tract (Yagishita et al., 1994). Even though ALS is primarily a motor neuron disorder, previous studies (Abrahams et al., 1996, 2005; Chang et al., 2005; Mezzapesa et al., 2007; Sage et al., 2007) have demonstrated involvement of extramotor regions subserving cognition and behavior, especially in ALS patients with dementia. Unlike Alzheimer’s dementia, cognitive impairment in ALS patients with dementia predominantly affects frontotemporal regions of the brain and is termed frontotemporal dementia (FTD).

Unlike LMN abnormalities, which can be identified by routinely used electromyography (EMG), even if such signs are subclinical, objectively identifying UMN abnormalities (Brooks et al., 2000) is more challenging. Techniques applied to assess the latter such as transcranial magnetic stimulation and proton magnetic resonance spectroscopy are more labor intensive, and primarily used in research settings (Kaufmann et al., 2004). The neurologic examination remains the gold standard for detecting UMN abnormalities, but this is relatively subjective and dependent on the skill and acumen of the clinician. If in contrast, LMN changes like muscle atrophy, hypotonia, and hyporeflexia are very prominent, coexistent UMN signs can be masked, making diagnosis of ALS very difficult. Therefore, we evaluated conventional neuroimaging techniques used during routine clinical evaluation to provide non-invasive objective measures of UMN involvement.

The focus of this review is to summarize our previously published findings of how non-biased conventional MRI sequences acquired at 1.5T have identified differences between ALS patients with specific clinical phenotypes. Our goal was to demonstrate the utility of widely accessible routine clinical MRI in revealing unique macropathologic differences *in vivo* between such ALS patient groups and possibly to gain insights into disease pathogenesis and progression.

## Patient Data Considered

Groups of individuals evaluated by conventional clinical T2/PD/FLAIR-weighted MRI included: (1) UMN-predominant ALS patients *with* CST hyperintensity (ALS-CST+), (2) UMN-predominant ALS patients *without* CST hyperintensity (ALS-CST-), (3) patients with classic ALS (ALS-Cl), and (4) ALS patients with frontotemporal dementia (ALS-FTD), and (5) neurological controls. UMN-predominant ALS patients were defined as those with LMN signs that were either absent, or if present, were restricted to only one neuraxial level (bulbar, cervical, or lumbosacral) at time of MRI. UMN-predominant patients with CST hyperintensity were those in whom hyperintense signal was observed along the CST bilaterally in T2-, FLAIR-, and especially PD-weighted images. Patients with ALS-FTD displayed cognitive or behavioral impairment during clinical evaluation, as assessed by EP Pioro, including disturbances of language, executive function and impulse control. Such patients underwent bedside evaluation, including MoCA testing, extensive formal neuropsychometric testing by an experienced neuropsychologist, and usually both.

## MRI Studies

**FIGURE 1:**
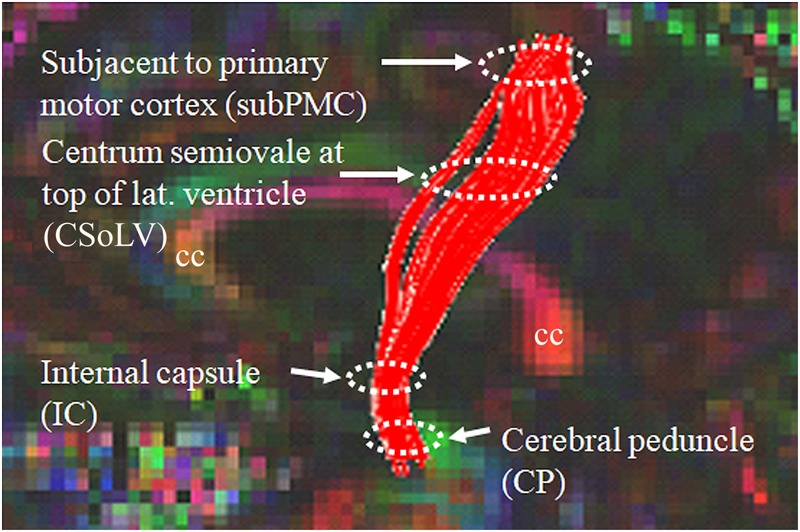
Sagittal view of control subject brain with superimposed FA color map showing tractography-derived virtual CST fibers between subjacent to primary motor cortex (subPMC) rostrally and cerebral peduncle (CP) caudally. DTI metrics are also obtained at two intervening CST levels, including centrum semiovale at top of lateral ventricle (CSoLV) and posterior limb of the internal capsule (IC). cc corpus callosum.

After identifying CST hyperintensity on T2-, FLAIR-, and PD-weighted sequences in several ALS patients with UMN-predominant phenotype, we were puzzled when we observed other patients, relatively indistinguishable at initial clinical evaluation, who did *not* have CST hyperintensity. Since diffusion tensor imaging (DTI) could provide more insight with its diverse metrics (which are based on diffusion of water molecules), we studied the DTI metrics: fractional anisotropy (FA), axial diffusivity (AD), radial diffusivity (RD), and mean diffusivity (MD) along the CST in ALS patients of ALS-CST+ and ALS-CST- groups, compared to neurologic controls (Rajagopalan et al., 2011). Four levels along the rostrocaudal extent of the CST (identified by diffusion tensor tractographic reconstruction) in the white matter (WM) were examined: (1) subjacent to primary motor cortex (subPMC), (2) centrum semiovale at top of lateral ventricle (CSoLV), (3) posterior limb of internal capsule (IC), and (4) cerebral peduncle (CP), as shown in Figure 1. This allowed us to determine in our UMN-predominant ALS patients the level(s) where abnormalities in DTI metrics occur along the CST. Furthermore, it enabled us to determine whether quantitative differences exist corresponding to the qualitative presence or absence of CST hyperintensity.

### Volumetric Studies in ALS-CST+ and ALS-CST- Patients

Our gray matter voxel based morphometry study (Rajagopalan and Pioro, 2014) revealed no significant difference in gray matter (GM) volume between ALS-CST+ and ALS-CST- groups in any brain region. Also, our brain parenchymal fraction (which includes GM and WM volume) study (Rajagopalan and Pioro, 2015) failed to reveal any significant difference in brain parenchymal fraction values between ALS-CST+ and ALS-CST- groups.

### DTI Metrics Distinguish Between ALS-CST+ and Other ALS Patient Groups

Fractional anisotropy values were reduced in both ALS-CST+ and ALS-CST- groups when compared to controls. On the other hand, the AD and RD metrics showed significant differences at the internal capsule level only between controls and the ALS-CST+ group but not in the ALS-CST- group. It is in the posterior limb of the IC that hyperintensity is usually reported in the ALS literature (Yagishita et al., 1994; Ellis et al., 1999; Toosy et al., 2003; Pioro, 2006). Considering that AD and RD metrics reflect axonal and myelin integrity (Beaulieu, 2009), their abnormality in the ALS-CST+ group but not the ALS-CST- group suggests micropathologic differences along the CST. These results suggest that ALS patients with CST hyperintensity probably have different underling pathology from those who do not, which could arise from differing pathogenic mechanisms.

**FIGURE 2:**
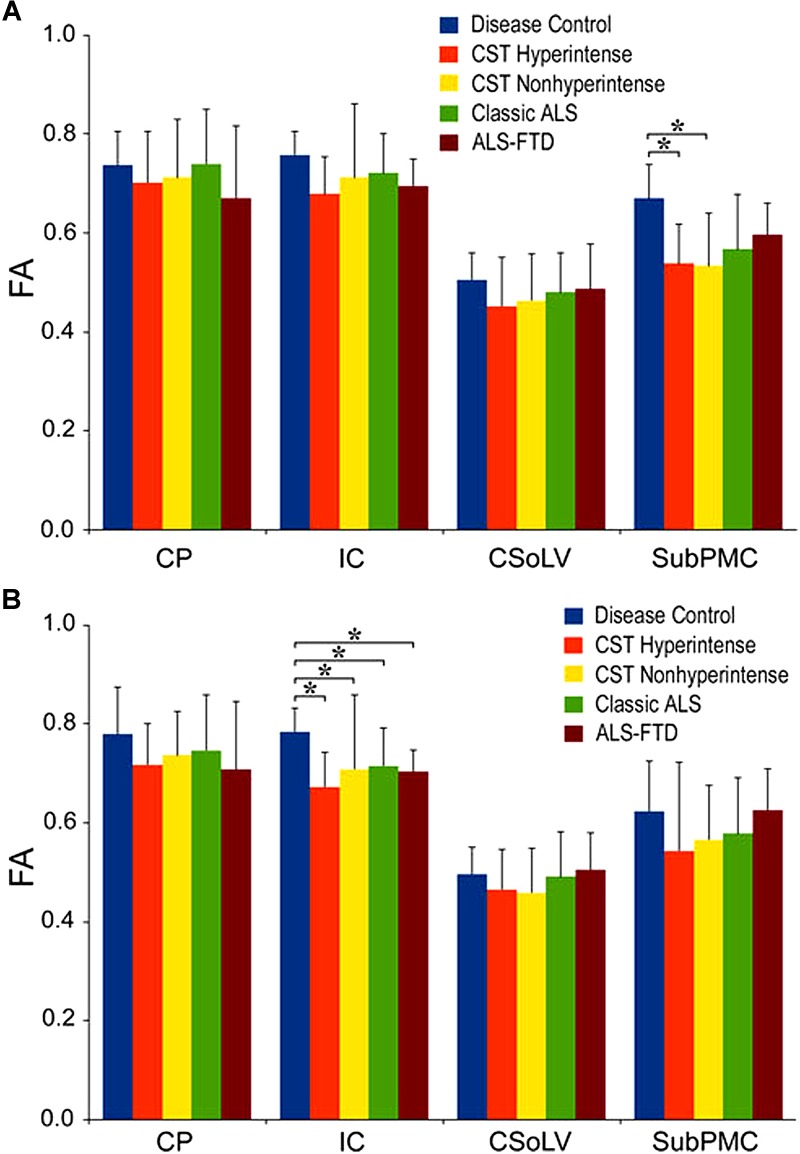
Fractional anisotropy (FA) values at four CST levels in left **(A)** and right **(B)** hemispheres of ALS patients compared to controls showing significant differences as ^∗^*P* < 0.05. CP, cerebral peduncle; IC, posterior limb of internal capsule; CSoLV, centrum semiovale at top of lateral ventricle; subPMC, subjacent to primary motor cortex. Reproduced with permission from Springer Nature.

**FIGURE 3:**
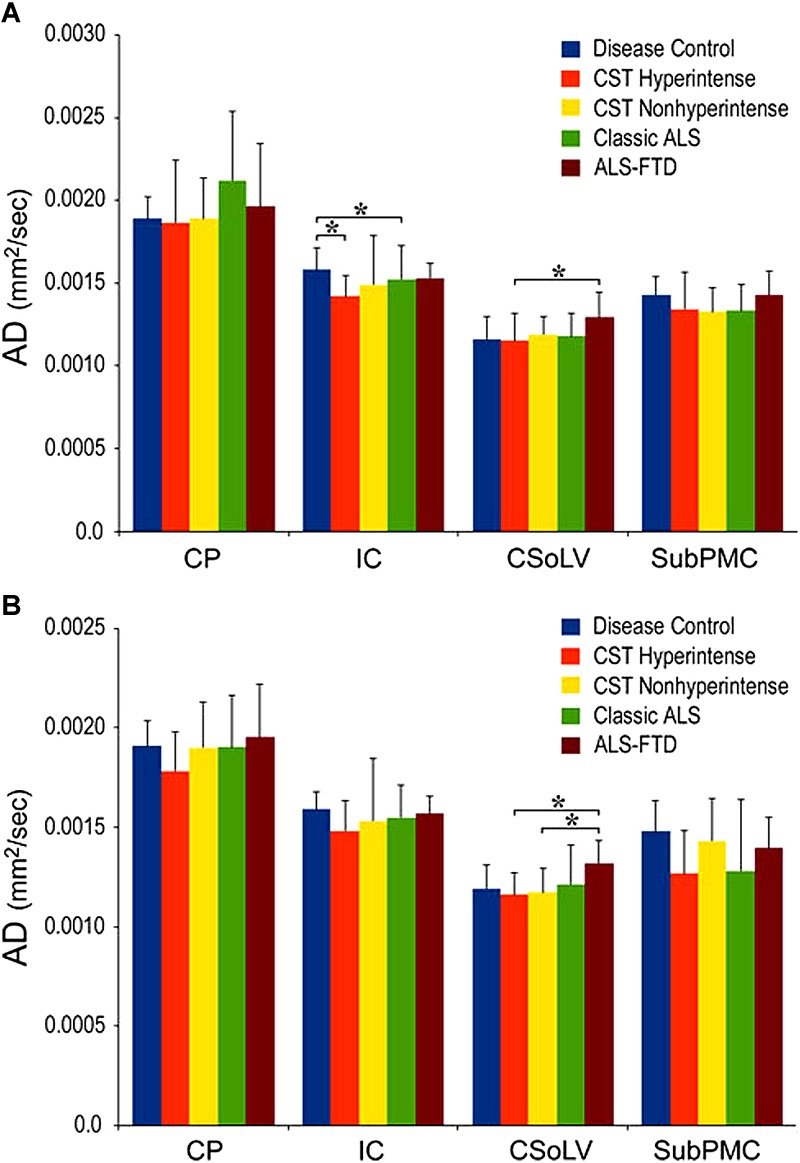
AD values at four CST levels in left **(A)** and right **(B)** hemispheres of ALS patients compared to controls showing significant differences as ^∗^*P* < 0.05. CP, cerebral peduncle; IC, posterior limb of internal capsule; CSoLV, centrum semiovale at top of lateral ventricle; subPMC, subjacent to primary motor cortex. Reproduced with permission from Springer Nature.

**FIGURE 4:**
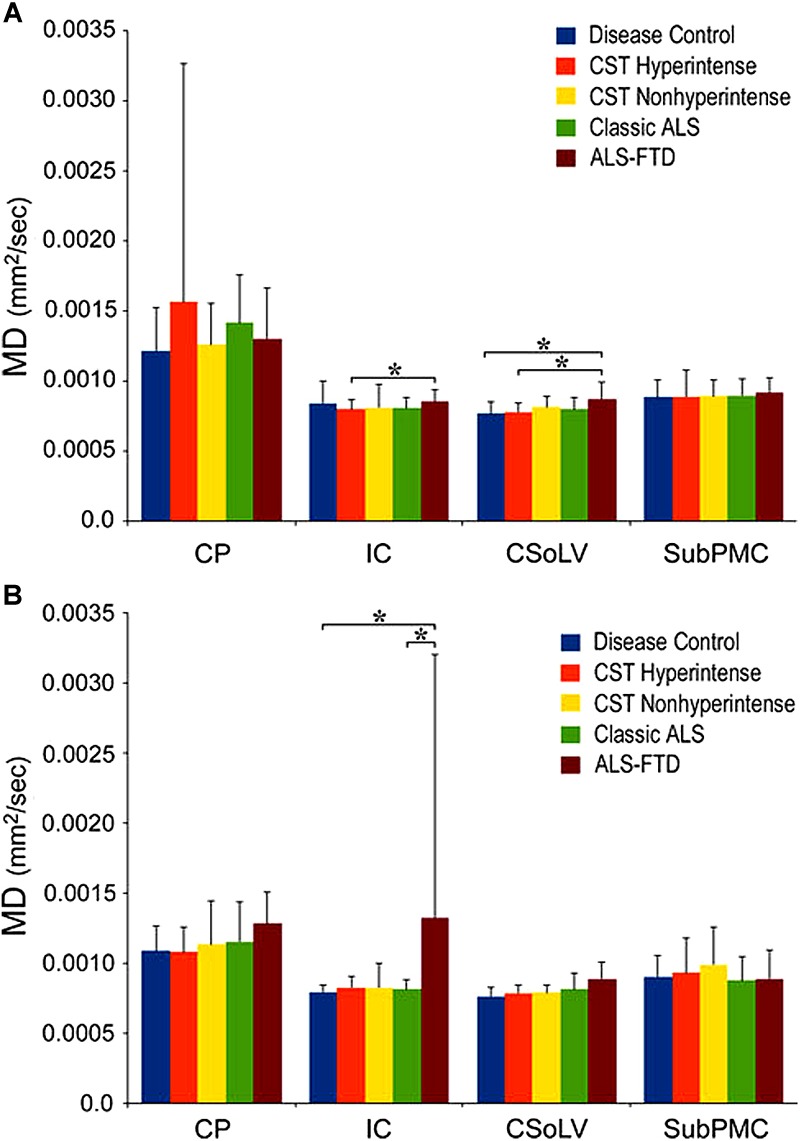
Mean diffusivity (MD) values at four CST levels in left **(A)** and right **(B)** hemispheres of ALS patients compared to controls showing significant differences as ^∗^*P* < 0.05. CP, cerebral peduncle; IC, posterior limb of internal capsule; CSoLV, centrum semiovale at top of lateral ventricle; subPMC, subjacent to primary motor cortex. Reproduced with permission from Springer Nature.

**FIGURE 5:**
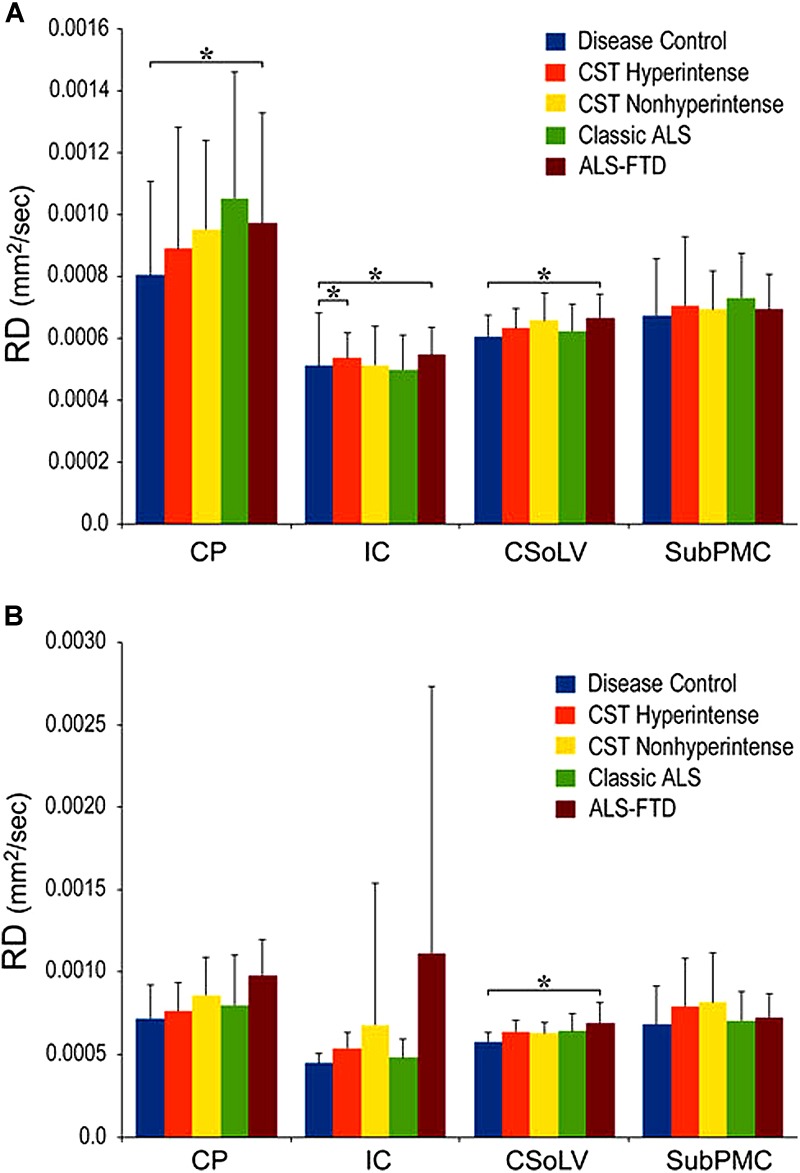
Radial diffusivity (RD) values at four CST levels in right **(A)** and left **(B)** hemispheres of ALS patients compared to controls showing significant differences as ^∗^*P* < 0.05. CP, cerebral peduncle; IC, posterior limb of internal capsule; CSoLV, centrum semiovale at top of lateral ventricle; subPMC, subjacent to primary motor cortex. Reproduced with permission from Springer Nature.

We further investigated whether neuroimaging, and specifically DTI metrics along the CST could objectively differentiate the ALS-CST+ group from the other ALS subtypes and neurologic controls (Rajagopalan et al., 2013b). In this study, we found that FA and AD values were lowest in the ALS-CST+ group when compared to controls and also when compared to the other ALS groups at rostral CST levels. When considering the CST separately in each hemisphere, significant FA differences were observed between controls and both ALS-CST+, and ALS-CST- groups (Figure 2). These findings, as well as significant differences in AD values between controls and patients in the ALS-CST+ group (but not between neurologic controls and those in ALS-CST- group) suggest differing micropathologies in the subcortical axons of the various ALS patient groups (Figure 3). Furthermore, AD, MD, and RD measures were significantly different between the ALS groups, distinguishing these values at IC and CSoLV levels of CST between patients in ALS-CST+ and ALS-FTD groups (Figure 3–5).

### Diffusion Tensor Tractography Reveals Motor Fiber-Specific Truncation

**FIGURE 6:**
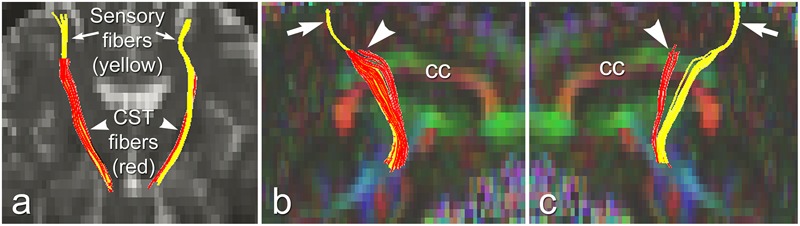
Truncated virtual CST fibers arising from primary motor cortex (red, arrowheads) are contrasted with intact sensory fibers projecting to/from primary sensory cortex (yellow, arrows) in an UMN-predominant ALS patient with faster disease progression rate. Virtual tracts are projected on a coronal b0 image **(a)**, and on sagittal images of left **(b)**, and right **(c)** hemispheres. cc, corpus callosum. Reproduced with permission from Elsevier.

The above studies demonstrated distinct pathological changes in regions of interest (ROIs) along the CST in the ALS-CST+ group compared to other ALS groups and neurologic controls. However, the ROI approach is limited because of operator bias where voxels are placed, and evaluation of the CST only where voxels are placed, rather than along its entire length. In order to more accurately and objectively identify areas along the tract’s entire length, we used diffusion tensor tractography (DTT) to reconstruct a “virtual” CST (Rajagopalan and Pioro, 2017).DTT identified virtual CST fibers between the CP and just beneath (Sub) the primary motor cortex (PMC) in ALS-CST+ patients, ALS-CST- patients, and neurologic controls. Surprisingly, we observed partial *absence* of virtual CST fibers in both groups of ALS patients but not in any controls. Specifically, these fibers were absent (“truncated”) at the SubPMC level, which is between the PMC and CSoLV levels in several patients of both ALS-CST+ and ALS-CST- groups, as shown from a representative patient in Figure 6. Of note, no truncation was observed in any of the neurologic control subjects. CST truncation occurred primarily in ALS-CST+ patients (9 of 21, 42.8%) and less frequently in ALS-CST- patients (4 of 24, 16.6%; *P* = 0.049). Further, the frequency of virtual CST truncation was significantly (*P* = 0.018) higher in all ALS patients (both ALS-CST+ and ALS-CST- groups combined) than in the control group. To determine if this truncation was specific to descending motor fibers, we identified virtual non-motor fiber tracts connecting the primary sensory cortex (PSC) and subcortical white matter. Because most of these sensory fibers are afferents to the PSC, they should generally be unaffected by corticomotoneuron degeneration. In fact, truncation of such virtual non-motor (sensory) tracts occurred in only one subject from each of the ALS patient groups: 1 of 21 (4.7%) in ALS-CST+, and 1 of 24 (4.1%) in ALS-CST- groups. Our DTT findings of subcortical truncation of essentially only virtual motor (and not sensory) fibers, as shown from a representative patient in Figure 6, suggest microanatomic specificity of the underlying pathophysiologic process. This is in keeping with the notion that the sensory system remains relatively unaffected in ALS with the minority of patients reporting sensory symptoms.

Importantly, the truncation of motor fibers more frequently in one ALS phenotype than another, no truncation in neurologic controls, and differential involvement of motor but not sensory fibers all suggest that virtual CST truncation is and disease- and fiber-type specific. Therefore, these results further support unique pathologies along the CST in these two UMN-predominant ALS patient groups.

### Disease Progression Rates Differ in Patients of ALS-CST+ and ALS-CST-Groups

Differences in the DTT findings between the two groups are supplemented by clinical observations of significantly shorter duration of symptoms prior to MRI in the ALS-CST+ group (9.6 ± 5.5 months, mean ± SD) compared to ALS-CST- group (36.4 ± 44.2 months, *P* < 0.001), as previously reported (Rajagopalan and Pioro, 2017; Table 1). The shorter disease duration in patients of the ALS-CST+ group compared to those of the ALS-CST- group translated into much faster disease progression in the former patients, even though both groups had essentially identical motor function scores of the revised ALS functional rating scale (ALSFRS-R) at time of MRI (34.6 ± 7.8, mean ± SD, vs. 34.1 ± 8.1, respectively). The monthly decline in ALSFRS-R (ΔFS) was three time higher in the ALS-CST+ group (1.38 ± 1.64, mean ± SD) compared to the ALS-CST- group (0.46 ± 0.43; *P* = 0.001), indicating a significantly faster decline of motor function in the former group of patients. Of note, duration of disease in the ALS-CST- group averaged 3 years prior to MRI (36.4 ± 44.2 months, mean ± SD), reflecting their slow progression, and was over 48 months in a one-third of them. This suggests that some of these slowest progressing patients without CST hyperintensity may have, in fact, represented a group with primary lateral sclerosis (PLS) (Gordon et al., 2006).

**Table 1:** Clinical parameters of ALS patients.

Clinical measure/ALS	ALS-CST+	ALS-CST-	
subgroups	Mean ± SD	Mean ± SD	*P*
*n*	21	24	NS
Age	52.3 ± 11.4	59.5 ± 12.1	<0.05
Symptom duration prior to	9.6 ± 5.5	36.4 ± 44.2	<0.001
MRI (months)
ALSFRS-R score	34.6 ± 7.8	34.1 ± 8.1	NS
Disease progression rate	1.38 ± 1.64	0.46 ± 0.43	0.001

*Key: SD, standard deviation. ALS-CST+, ALS patients with predominant upper motor neuron (UMN) signs and hyperintense signal along the corticospinal tract (CST) on conventional proton density (PD) and T2-weighted images and no clinical dementia. ALS-CST-, ALS patients with predominant UMN signs without CST hyperintensity and no clinical dementia (ALS-CST-). ALSFRS-R, ALS functional rating score-revised. NS, not significant.*

### Fractal Dimension Analyses Reveal ALS Group Differences in White Matter Complexity

At a microscopic level, ALS pathology includes axonal swelling with neurofilament accumulation, dendritic attenuation, and wallerian degeneration of axons (Cluskey and Ramsden, 2001). Evidence of such micropathology, including axon degeneration and demyelination can be detected at a macroscopic level *in vivo* by certain MRI techniques (Metwalli et al., 2010). Neuronal degeneration with resultant loss of dendrites and axons has been shown to reduce complexity of subcortical WM structure. Therefore, measuring WM structural complexity may reveal the effects of neuronal degeneration occurring in ALS.

Fractals are geometry objects that are self-similar at different scales, and were first proposed by Mandelbrot. The fractal dimension (FD) is a non-integer number that characterizes the morphometric variability of a complex and irregular shape. FD analysis can quantitatively measure the internal shape complexity of brain WM from MRI by characterizing multifractal behavior of different textures instead of using only pixel intensity values (Liu et al., 2003). Higher FD values reflect more WM complexity, as would be expected in healthy states, whereas lower values result with aging, and when WM becomes diseased more amorphous. In a study of patients with multiple sclerosis, reduced brain WM FD values were proposed to represent a more amorphous tissue state resulting from inflammation, decreased myelin content, and increased water content (Esteban et al., 2007).

**FIGURE 7:**
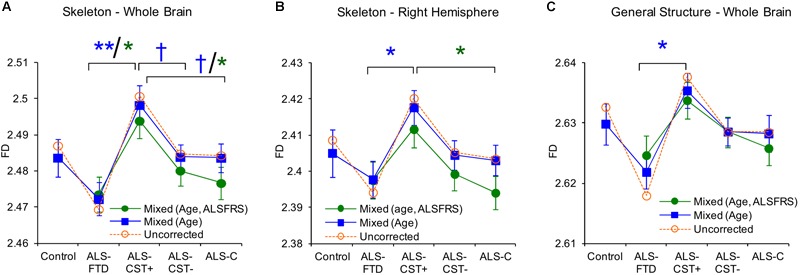
Between group comparisons show significant differences in fractal dimension values of skeleton-whole brain **(A)**, skeleton right hemisphere **(B)**, and general structure whole brain **(C)**. Uncorrected means are represented as dashed lines and corrected means (mixed model) and standard error of the mean are shown as solid lines. Data for mixed models with gender, age as covariates are shown in blue or with gender, age, and ALSFRS-R as covariates are in green. Corrected mean comparisons between groups are performed using the Tukey multiple comparison method. ^†^*p* < 0.1, ^∗^*p* < 0.05, ^∗∗^*p* < 0.001. Reproduced with permission from *PlosOne*.

We used FD analysis to evaluate WM structural degeneration in each of the four ALS patient groups: ALS-CST+, ALS-CST-, ALS-Cl, and ALS-FTD. In this study (Rajagopalan et al., 2013a), we estimated three quantitative measureable WM features using FD shape representations, including WM skeleton, GM/WM surface structure, and WM general structure. The skeleton captures the central line of the WM structure, which preserves the topological and geometric information of the WM, and represents its interior structure complexity. The surface structure comprised of voxels at the gray matter (GM)-WM interface, represents the shape of gyral and sulcal convolutions over the cortical surface. Finally, FD of general structure incorporates all WM voxels, including those at the GM/WM interface and skeleton in segmented images, and thereby represents brain volume. Because FD measures of skeleton, surface structure, and general structure represent different components of WM, they provided novel information about ALS-induced changes in brain WM structure and shape. General structure and skeleton FD values were significantly different between ALS-CST+ and ALS-FTD groups. Whole brain skeleton (*P* = 0.001) and general structure (*P* = 0.02) were significantly higher in ALS-CST+ patients compared to ALS-FTD patients, as shown in Figure 7. Although not significant, whole brain skeleton FD values in ALS-CST+ group patients trended higher than those in ALS-CST- (*P* = 0.10) and ALS-Cl (*P* = 0.10) groups. However, neurologic controls and ALS patients revealed no significant differences in FD values. These results indicate that shape complexity in the ALS-CST+ patient group was significantly greater than in the ALS-FTD group, and trended higher than in the other two patient subtypes. Although the significance of this higher FD in ALS-CST+ patients is unclear, it is likely related to differences in integrity of axons, myelin, and other changes within the neuropil, including inflammatory processes.

### Clinical Differences Between Patients in ALS-CST+ and Other Groups

Patients in the ALS-CST+ group were younger when compared to those in the ALS-CST- group (*P* < 0.05) (Table 1). In contrast, patients with ALS-FTD were significantly older than ALS-CST+ patients and neurologic controls. ALS-CST+ group revealed significantly shorter symptom duration compared to those in ALS-CST- (*p* < 0.001) and ALS-FTD groups (*p* < 0.05), indicating earlier neurologic evaluation after symptom onset. Also, disease progression rate was significantly faster in ALS-CST+ patients than in ALS-CST-, ALS-Cl, and ALS-FTD groups, as had been observed in a preliminary study of another group of ALS patients (Matte and Pioro, 2010). The revised ALSFRS-R score, which is a validated measure of physical function in ALS (Cedarbaum et al., 1999), was significantly lower (worse) in ALS-FTD patients compared to the ALS-Cl patients but essentially identical in ALS-CST+ and ALS-CST- patients at time of MRI.

## Conclusion

The aforementioned brain MRI studies uniformly revealed objective differences in patients with the various ALS subtypes: UMN-predominant ALS with CST hyperintensity (ALS-CST+), UMN-predominant ALS without CST hyperintensity (ALS-CST-), classic ALS (ALS-Cl), and ALS with FTD (ALS-FTD). Specifically, patients in the ALS-CST+ group show distinctive and distinguishable changes from the others, including patients in the ALS-CST- group, which appear phenotypically similar, at least in relation to extent of UMN dysfunction. Coupled with the patients’ distinct clinical characteristics, these neuroimaging abnormalities strongly suggest that CST hyperintensity, as revealed by conventional MRI (cMRI) T2/PD, and FLAIR sequences used during routine clinical evaluation, is not artefactual or non-specific but identifies a unique ALS patient group. *We hypothesize that ALS-CST+ patients comprise a distinct phenotype from ALS-CST-, ALS-Cl, and ALS-FTD with unique micropathology of the CST and potentially important differences in ALS pathogenesis.* Prescreening ALS patients for the presence of CST hyperintensity may be useful when enrolling or stratifying into clinical trials.

## Author Contributions

VR and EPP conceptualized the idea for the manuscript. VR wrote the manuscript. EPP made substantive revisions to the manuscript.

## Conflict of Interest Statement

The authors declare that the research was conducted in the absence of any commercial or financial relationships that could be construed as a potential conflict of interest.
